# The Nerves of the Heart

**Published:** 1869-08

**Authors:** 


The Nerves of the Heart.—Dr. Cyon has been just awarded, by the Imperial Academy of Sciences, the annual premium for discoveries in experimental physiology, for the discovery of two nerves going from the spinal marrow to the heart, and belonging properly to that organ. His paper is entitled "Researches on the Innervation of the Heart by the Spinal Marrow," and, besides, a description of the newly-discovered nerves, relates demonstrations of new phenomena, produced by these nerves in the functions of the important organ to which they belong. The first nerve, which he calls the special cardiac accelerator nerve, emerges from the spinal column with the third branch of the cervical ganglion, and, when excited on the living animal, the heart's pulsations are increased. The same effect is produced whether the excitation is made on the root of the nerve, or on the spinal marrow near the nerve. The other nerve discovered by M. Cyon, uniting the heart to the spinal marrow, is not a motor but a sensitive nerve. By the medium of this nerve a reflex action is produced, starting from the heart, and influencing the capillary circulation in all the organs of the body. The most important facts in the physiology and pathology of the heart may flow from this discovery. M. Claude Bernard is now at work at the new nerves, and some prominent indications have been established, or nearly so, relative to disease of the heart.—*Paris Correspondent of the Times.*

